# Analyze Informant-Based Questionnaire for The Early Diagnosis of Senile Dementia Using Deep Learning

**DOI:** 10.1109/JTEHM.2019.2959331

**Published:** 2019-12-16

**Authors:** Fubao Zhu, Xiaonan Li, Daniel Mcgonigle, Haipeng Tang, Zhuo He, Chaoyang Zhang, Guang-Uei Hung, Pai-Yi Chiu, Weihua Zhou

**Affiliations:** 1 School of Computer and Communication EngineeringZhengzhou University of Light Industry 117776 Zhengzhou450002China; 2 School of Computing Sciences and Computer EngineeringUniversity of Southern Mississippi 5104 Long BeachMS39560USA; 3 College of ComputingMichigan Technological University 3968 HoughtonMI49931USA; 4 Department of Nuclear MedicineChang Bing Show Chwan Memorial Hospital 92748 Changhua505Taiwan; 5 Department of NeurologyShow Chwan Memorial Hospital 63295 Changhua500Taiwan

**Keywords:** Dementia, information gain, deep neural network, machine learning

## Abstract

Objective: This paper proposes a multiclass deep learning method for the classification of dementia using an informant-based questionnaire. Methods: A deep neural network classification model based on Keras framework is proposed in this paper. To evaluate the advantages of our proposed method, we compared the performance of our model with industry-standard machine learning approaches. We enrolled 6,701 individuals, which were randomly divided into training data sets (6030 participants) and test data sets (671 participants). We evaluated each diagnostic model in the test set using accuracy, precision, recall, and F1-Score. Results: Compared with the seven conventional machine learning algorithms, the DNN showed higher stability and achieved the best accuracy with 0.88, which also showed good results for identifying normal (F1-score = 0.88), mild cognitive impairment (MCI) (F1-score = 0.87), very mild dementia (VMD) (F1-score = 0.77) and Severe dementia (F1-score = 0.94). Conclusion: The deep neural network (DNN) classification model can effectively help doctors accurately screen patients who have normal cognitive function, mild cognitive impairment (MCI), very mild dementia (VMD), mild dementia (Mild), moderate dementia (Moderate), and severe dementia (Severe).

## Graphical abstract

We present a deep neural network (DNN) classification model that can help clinicians accurately screen patients who have normal cognitive function, mild cognitive impairment (MCI), very mild dementia (VMD), mild dementia (Mild), moderate dementia (Moderate), and severe dementia (Severe).

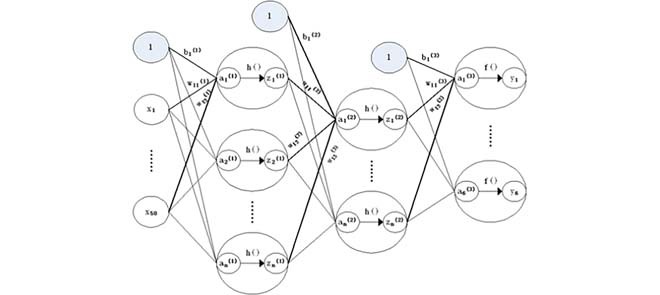

## I. Introduction

Dementia characterized by cognitive and intellectual impairment is a kind of neurodegenerative diseases [1]. Dementia diagnosis is a critical issue since it affects 47.5 million people worldwide according to World Health Organization [2]. Currently, cognitive and/or memory disorders are the primary metrics [3] used to identify whether individual suffers the dementia or not. To assess the cognitive status of a patient the neuropsychological tests are commonly used in clinical diagnosis. Nevertheless, it is time-consuming for the manual diagnosis of cognitive impairment by using neuropsychological tests. Moreover, the efficiency and accuracy of the diagnosis are determined by the professional level of the practitioner. In some remote areas lacking professional personnel, it will be a much more difficult task for classification and the early diagnosis of dementia.

In recent years, deep neural network (DNN) has garnered clinical interests in cognitive diagnostic applications due to its advantages in efficient classification. Moreover, many existing methods have been proposed, where some of them [4]–[7] combine DNN with neuroimaging markers, while other methods [8]–[10] combine DNN with neuropsychological assessments. Jain et al. [5] proposed a transfer learning approach for accurately classifying brain sMRI slices amongst 3 different classes: Alzheimer’s disease (AD), cognitively normal(CN) and mild cognitive impairment (MCI). For the validation set, the accuracy of the three-way classification using their method was 95.73%. However, they only analyzed the T1-weighted sMRI data of 150 subjects/patients. Lu *et al.* [6] proposed a novel deep-learning-based framework to discriminate individuals with AD utilizing a multimodal and multiscale deep neural network. They obtained an accuracy of 82.4% in identifying the individuals with MCI. They analyzed both a T1-weighted MRI scan and FDG-PET image data of 1242 subjects/patients. Orimaye *et al.* [8] proposed a method that combined deep neural network and deep language models (D2NNLM) for classifying the disease. The experimental results showed that the model could accurately predict MCI and AD type dementia on a very sparse clinical language dataset. Themistocleous et al. [10] provided an automated deep learning method using DNN architectures that identified individuals with MCI from healthy controls. However, there are still limitations in the current studies. First, the amount of data used in the methods introduced above was not sufficient. This limitation may lead to a decrease in the reliability of experimental results. Second, these methods focused more on binary classification problem. Yet, as dementia is currently irreversible and incurable, multi-class classification for different dementia stages is actually of much more clinical interest.

Hence, we propose a DNN multi-class classification model to assist the preliminary diagnosis of normal, mild cognitive impairment (MCI), very mild dementia (VMD), mild dementia (Mild), moderate dementia (Moderate), and severe dementia (Severe) using informant-based questionnaire. In this paper, 6,701 individuals are enrolled, which allow us to have a larger size of samples to train our model.

## II. Materials and Methods

There are two major steps in the proposed framework: (1) feature selection: using feature selection algorithms to optimize or even reduce the number of neuropsychological tests; (2) classification: training a deep neural network to classify the participants into normal cognitive function, MCI, VMD, Mild, Moderate, and Severe categories.

### A. Patient Sample Collection

In this work, the study used data collected from the three centers of the Show Chwan Healthcare System. The data selected from the register-based database of the Show Chwan Health System were analyzed anonymously with the informed consent from all participants, and the study was designed retrospectively in accordance with relevant guidelines and regulations. The project was reviewed by the Medical Research Ethics Committee of Show Chwan Memorial Hospital, and the study was approved by the Data Inspectorate.

The data for the study consisted of samples of clinical and neuropsychological assessment obtained from 6701 patients. For detailed neuropsychological tests, we assessed the history of cognitive status and objective assessments including the Clinical Dementia Ratings (CDR), Mini Mental Status Examination (MMSE), Cognitive Abilities Screening Instrument (CASI) and Montreal Cognitive Assessment (MoCA) performed to evaluate memory, executive function, orientation, visual-spatial ability, and language function [11]. Along with the current scales such as CDR, MMSE, CASI, MoCA, we used a newly designed Informant-based questionnaire named HAICDDS which is applied in dementia registration in a health system with 9 regional hospitals in Taiwan. Clinical application of the HAICDDS had been published in journals [11]–[13] or conferences [14], [15]. The CDR determined the severity of dementia. Experienced neurologists evaluated the participants based on their clinical symptoms and reviews of medical/medication history, neuropsychological test results, and then classified the participants into six diagnostic groups: normal (535 participants), MCI (1687 participants), VMD (678 participants), Mild (1812 participants), Moderate (1309 participants), and Severe (680 participants). The six diagnostic groups were defined using the CDR staging. Among CDR 0.5, participants without significantly impaired activities of daily living were divided as CDR 0.5 MCI and those with significantly impaired activities of daily living were divided as CDR 0.5 VMD. Therefore, the 6 groups were CDR 0, CDR 0.5 MCI, CDR 0.5 VMD, CDR 1, CDR 2, and CDR 3. The operational diagnosis of a significant interfere with ADL is the IADL total score <7.

we randomly split the data with the ratio of 9:1, of which 90% are training data sets (6030 participants) and 10% are test data sets (671 participants) [16]. In order to estimate the generalization error, this procedure was repeated 10 times independently to avoid any deviation caused by randomly partitioning data sets. The average accuracy and F1-score were calculated for performance analysis. We finally obtained 10 training-test of different training set (6030 participants) and test set (671 participants). We also repeated the independent training-test procedure more than 10 times (k = 10) but the results were similar, so only the results with k = 10 were reported in the manuscript.

### B. Feature Selection

Neuropsychologists selected 50 items from neuropsychological tests to form an optimal questionnaire for screening patients with varying severities of dementia. While the proposed algorithm still works with the entirety of those features, utilizing feature selection lowers the computational requirements and improves interpretability as we are able to see more clearly which items are directly correlated with a patient’s mental condition [11]. In order to retain the features with higher prediction performance, provide faster and more cost-effective predictors, reduce the curse of dimensionality problem and the possibility of overfitting during the training phase, we used information gain feature selection algorithms to rank the importance score of all 50 features and then the low ranking features were filtered out. We discard features with a lower score one by one, and input the remaining features into the DNN model to observe the change of the classification accuracy in order to identify the feature set with a smaller number of features but only a minor drop-off of classification accuracy.

Information gain is an information theory method widely used in data mining. The information gain measures how much information the feature can provide the classification model. If a feature has a larger information gain value for a class, the feature contains more classification information for that class. We used the information gain algorithm provided by Weka, which is an open source machine learning and data mining software based on the Java environment.

### C. Overview of Methods

We proposed a DNN classification model based on the Keras framework. In order to study the performance of the DNN model for discriminating normal, MCI, VMD, Mild, Moderate, and Severe, we compared its results with others well-known classification models (MLP, GCForest, random forest, AdaBoost, LogitBoost, Naïve Bayes and SVM). First, we tested the performance of the model respectively using 50 features selected by the Neuropsychologists and the top 44 features selected by information gain score. Then, for testing the stability of the classification model, we tested 10 training-test runs separately. Finally, we evaluated the accuracy of model classification by measuring the average accuracy of the 10 training-test runs and evaluated the classification performance of each sub-category by using the 10 training-test runs with the lowest F1-score. TP is the number of positive samples predicted by the classifier in the number of true positive samples, FP is the number of positive samples predicted by the classifier in the number of true negative samples, TN is the number of negative samples predicted by the classifier in the number of true negative samples, FN is the number of true positive samples predicted by the classifier as negative samples. The accuracy is the evaluation of the correct rate of the classifier as a whole. It is defined as Accuracy = (TP+TN)/(TP+FN+FP+TN). Generally speaking, the higher the accuracy, the better the classifier. F1-Score is a kind of statistic, which is also called F-measure. F1-Score is the weighted harmonic average of Precision and Recall(Sensitivity). It is defined as F1-Score = 2TP/(2TP+FP+FN) and carries a range of 0 to 1, with higher scores indicating a more robust classification model. It is a commonly used evaluation criterion in the field of IR (Information Retrieval). It is often used to evaluate the quality of classification models. Precision, also called Positive Predictive Value in clinical settings, refers to how many of the samples that the model is positive are true positive samples, which is defined as Precision = TP/(TP+FP). Recall, also called Sensitivity in clinical settings, refers to how many positive samples are classified as positive by the model, which is defined as Recall = TP/(TP+FN).

#### 1) DNN

DNN is a multi-hidden layer feedforward neural network, which has a total of L+1 layers, the 0th layer is the input layer, the 1st to L−1 layers are hidden layers, the Lth layer is the output layer. The nodes of adjacent layers are connected by links and the weights of all links form a feedforward weight matrix. As shown in Eqs.(1)–(2), suppose there are neurons in the lth layer, and the input vector of these neurons is $a^{(l)}$, the output vector is $z^{(l)}$. At the same time, we distinguish the final output of DNN on the output of the hidden layer by u $=\,\,\text{y}^{(L)}$. Given the characteristic x of a training sample, there is $\text{a}^{(0)} =\text {z}^{(0)}=\text {x}$.

\begin{align*} a^{(l)}=&W^{(l)}\ast a^{(l-1)}+b^{(l)},\quad l= 1,2,\ldots \mathrm {L} \tag{1}\\ z^{(l)}=&h(a^{(l)})\tag{2}\end{align*}

where $\text{W}^{(l)}$ is the weight matrix of the l-1 layer to the lth layer, b is the offset vector of the lth layer, h() is the activation function of the lth layer.

**FIGURE 1.**
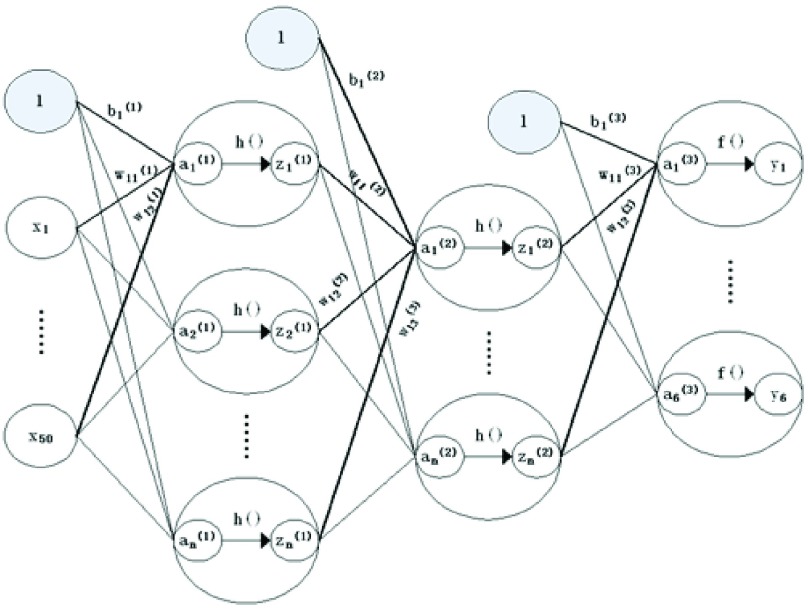
DNN model structure.

As a feedforward neural network, given an input vector, DNN can get an output vector immediately, that is to say, the output of DNN only depends on the current input, so DNN is suitable for pattern classification problem. This paper adjusts the network parameters of the whole network through supervised training. After repeated extensive training, we got relatively optimal hyperparameters in DNN. The constructed DNN model consists three hidden layers, the first layer uses the relu (rectified linear unit) activation function, the second layer uses the tanh [17] activation function, the third layer uses the softmax activation function. The epoch is set to 40, the dropout rate is set to 0.2, the batch size is set to 32, the learning rate is set to 0.004, and the number of neurons is set to 20 in each layer. The specific structure of the DNN model is shown in the figure 1 below. where $l$ is the number of layers, x is the input feature, b is the offset vector of the lth layer, $\text{W}^{(l)}$ is the weight matrix of the l-1 layer to the lth layer, $a^{(l)}$ is the input vector of the lth layer, h() is the activation function of the lth layer, $\text{z}^{(l)}$ is the input vector of the next layer, f () is the output activation function and y is the output vector.

#### 2) Other Investigated Classification Models

We investigated other commonly used classification models (MLP, GCForest, random forest, AdaBoost, LogitBoost, Naïve Bayes and SVM) in Python toolbox [16], which is a set of freeware academic software packages. The following briefly describes the basic principles of the classification models and the further details can refer to cited literatures.

An MLP can be seen as a directed graph, consisting of multiple node layers, each layer connected to the next layer. In addition to input nodes, each node is a neuron (or processing unit) with a non-linear activation function0. Unlike the DNN model, the output activation function is not used here. GCForest is a model of a deep forest, which is mainly divided into two parts, multi-grained scanning, and cascade forest structure. GCForest performs well in small sample data. Random forest is an algorithm that integrates multiple trees by the idea of ensemble learning. Its basic unit is a decision tree, which is a subclass of ensemble learning. It depends on the voting choice of a decision tree to determine the final classification results. In the basic Adaboost algorithm, each weak classifier has the right to weight, and the weighted sum of the weak classifier prediction results forms the final prediction result. In training, training samples have also weight, which dynamically adjusts during the training process. The samples that are misclassified by the previous weak classifier will increase the weight, so the algorithm will focus on the difficult samples. The Logitboost algorithm is a discriminant classification algorithm based on machine learning. LogitBoost belongs to the AdaBoost system. The LogitBoost structure is similar in general, but its loss function uses the maximum logarithmic likelihood function. The basic method of Naïve Bayes is to calculate the probability that the current feature samples belong to a certain classification based on the statistical data and the conditional probability formula, and select the maximum probability classification0. Support Vector Machine (SVM) has achieved the best performance in many classification problems. The kernel function subtly transforms the linear indivisible problem into a linear separable problem, and has very good generalization performance.

## III. Results

### A. Feature Analyses

We find that the classification accuracy decreases with the decrease of the number of features. When the number of features decreases to 44 features, the classification accuracy dropped down. So we discarded the corresponding features by setting the threshold of the information gain score to 0.16.

**FIGURE 2.**
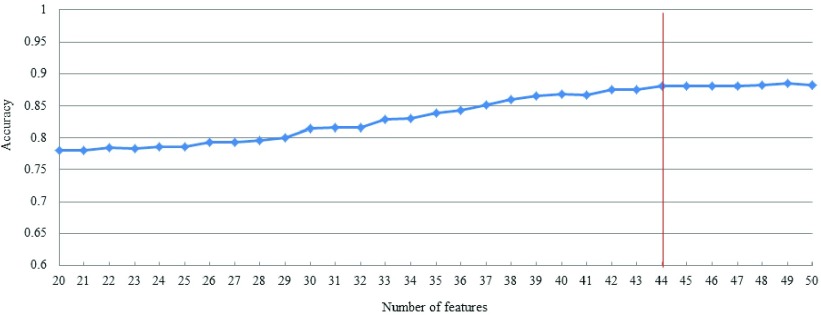
Classification accuracy with decreasing number of features.

Figure 2 shows the trend of classification accuracy by our DNN model as the features with lower scores are discarded one by one. The classification accuracy is the average of ten experiments. With a decreasing number of features, the classification accuracy decreases. After reducing to 44 features, the subsequent classification accuracy has declined by a certain extent.

**FIGURE 3.**
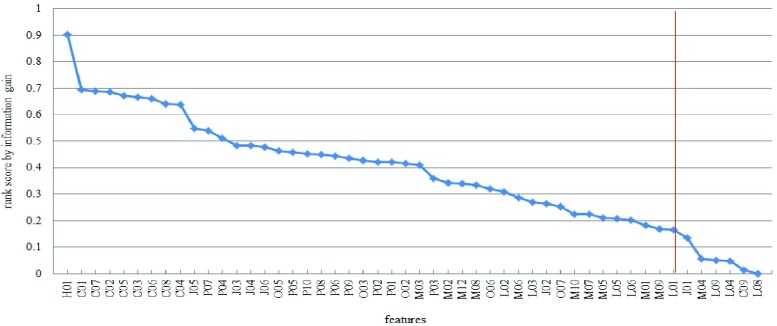
Features ranked according to their information gain scores.

Figure 3 shows the features ranked in descending significance with respect to the information gain scores. The cut-off is shown reducing the number of features to the 88% with setting the threshold of the information gain score to 0.16, thus reducing the feature number from 50 to 44 features. Among the top 44 selected features, the feature ‘H01’ has the highest ranking score of 0.902, and the feature ‘L01’ has the lowest ranking score of 0.1665.

### B. Performance of Classification Models

**FIGURE 4.**
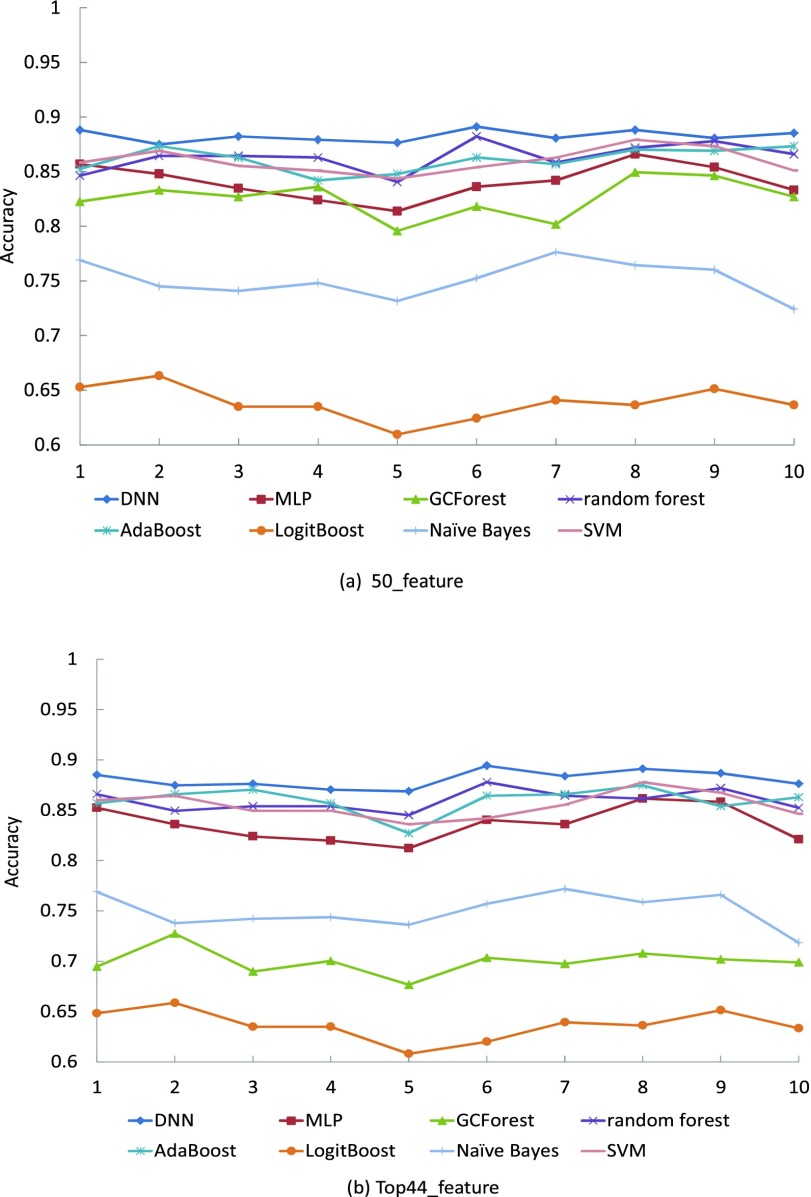
Performance of accuracy for each of the 10 rounds.

Figure 4 shows the classification performance for each of the 10 rounds individually. (a) shows the accuracy analysis results using 50 features selected by the Neuropsychologists. (b) shows the accuracy analysis results using the top 44 features selected by information gain score. The accuracy performance of the DNN reaches a plateau in the 10 rounds, which are better than other algorithms. The performance of the model reduces when lower features are used as input into the classifier.

**FIGURE 5.**
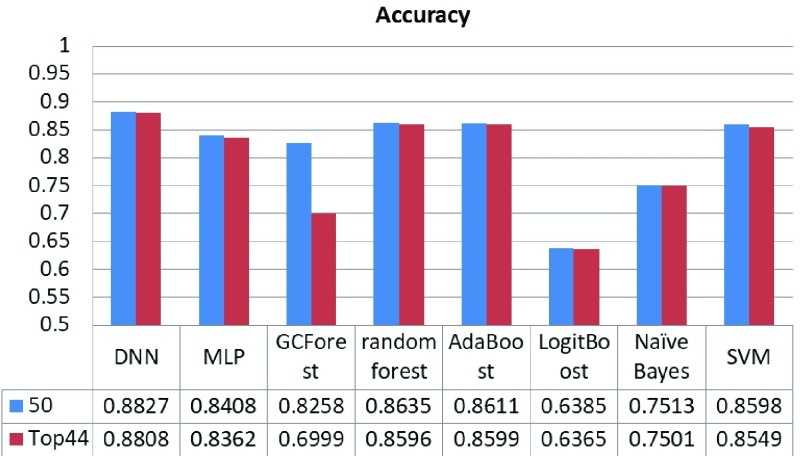
Comparison of the accuracy obtained by DNN and other classifiers.

Figure 5 shows the average results in 10 rounds of the comparison of our DNN accuracy performance results and other well-known classifiers (MLP, GCForest, random forest, AdaBoost, LogitBoost, Naïve Bayes and SVM) for the same dataset. When using 50 features, the best accuracy was obtained by the DNN classifier (accuracy = 0.8748), followed by the MLP classifier (accuracy = 0.851). When using the top 44 features, the DNN classifier performs the best (accuracy = 0.8808), followed by the AdaBoost (accuracy = 0.8599).

### C. Multi-Class Classification

**FIGURE 6.**
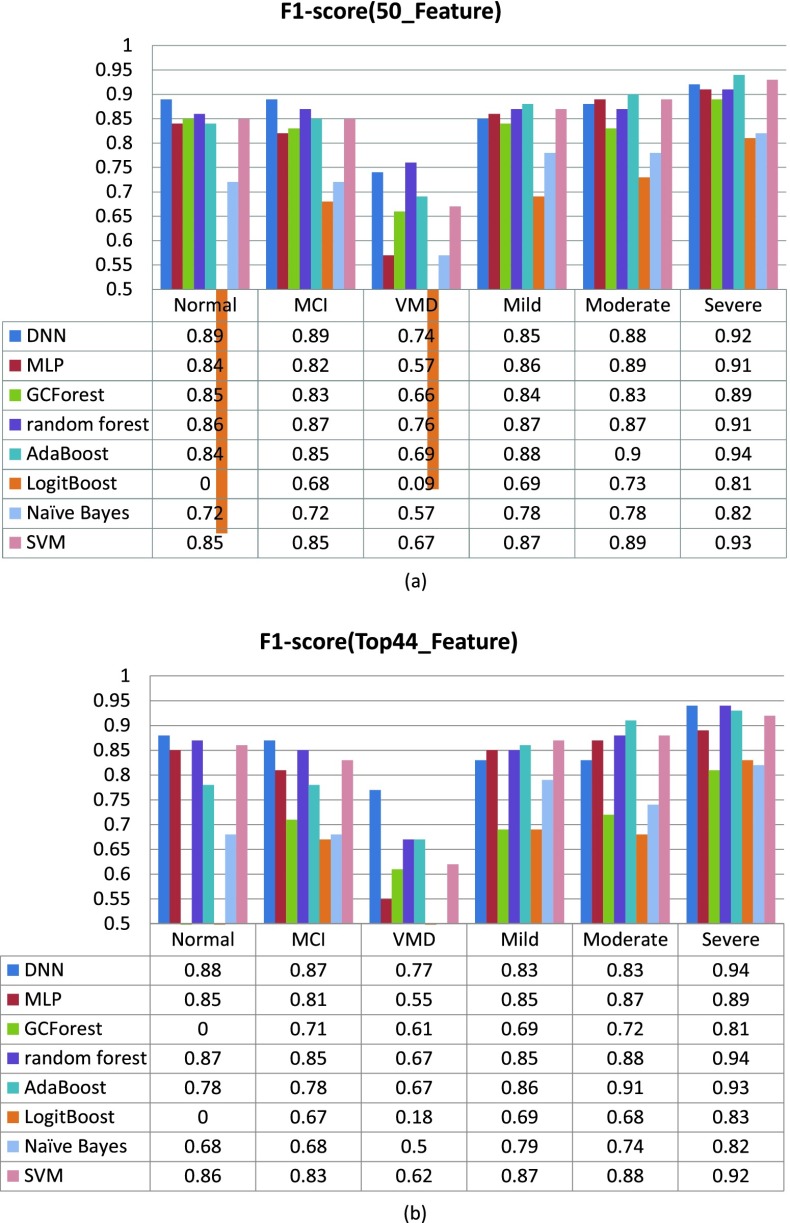
Performance of F1-score in the classification of normal, MCI, VMD, Mild, Moderate and Severe using classifiers.

Figure 6 compares the F1-score performance of the 10 rounds in the classification of normal, MCI, VMD, Mild, Moderate and Severe using DNN, MLP, GCForest, random forest, AdaBoost, LogitBoost, Naïve Bayes and SVM. As shown in Figure 6, when using all the 50 features, the DNN algorithm effectively improved the overall performance in classifying normal (F1-score = 0.89), MCI (F1-score = 0.89), VMD (F1-score = 0.74), Mild (F1-score = 0.85), Moderate (F1-score = 0.88) and Severe (F1-score = 0.92). When using the top 44 features selected by information gain score. The DNN algorithm performed best result in screening the normal (F1-score = 0.88), MCI (F1-score = 0.87), VMD (F1-score = 0.77) and Severe (F1-score = 0.94), and poorest in Mild (F1-score = 0.83) and Moderate (F1-score = 0.83) categories.

## IV. Discussion

In this study, we proposed a deep neural network classification model based on the Keras framework. In order to evaluate the advantages of our proposed method, we compared two indicators, accuracy and F1-score. In addition, we compared our method with other well-known machine learning methods. The results showed our DNN method had a stable classification performance, higher classification accuracy and performed well in dealing with class imbalance problems. It has great potential for clinical application. We will discuss these in detail below.

From the perspective of model classification stability and accuracy, when using 50 features by the Neuropsychologists, the DNN model shows higher stability and the classification accuracy is the highest compared with the other seven algorithms (MLP, GCForest, random forest, AdaBoost, LogitBoost, Naive Bayes and SVM), basically stable at around 0.88. When it comes to the classification accuracy of each category, our results show that the DNN model improved the overall performance of the classification accuracy of each category.

We further studied the classification performance of the DNN model after reducing features by information gain feature selection, which is to simplify the procedure of diagnosis and enhance the practicality in clinic. The overall classification performance of the model has decreased after the reduction. In order to ensure the classification accuracy of the model, we set the threshold of information gain fraction to 0.16, thus discarding some redundant features. Compared with the six classification models, the DNN performed the best accuracy with 0.88, which also showed good results for identifying normal (F1-score = 0.88), MCI (F1-score = 0.87), VMD (F1-score = 0.77) and Severe (F1-score = 0.94).

## V. Conclusion

We proposed a new approach to diagnosing normal, MCI, VMD, Mild, Moderate, and Severe using a deep learning approach, more specifically, a deep neural network classification model based on the Keras framework. By using the real-world dataset, i.e., the register-based database in the Show Chwan Health System, we tested and validated our method. Overall, the results of this project show that the proposed DNN model provides a tool with accurate and stable performance for clinicians to diagnose the early stages of dementia. Our future work will be carried out from neuroimaging to further improve our diagnostic model.
